# Editorial: The Elephant in the Room: AML Relapse After Allogeneic Hematopoietic Cell Transplantation

**DOI:** 10.3389/fonc.2022.863800

**Published:** 2022-03-21

**Authors:** Giorgia Battipaglia, Francesco Saraceni, Florent Malard

**Affiliations:** ^1^ Hematology Department, Federico II University of Naples, Naples, Italy; ^2^ Department of Clinical Medicine and Surgery, Federico II University of Naples, Naples, Italy; ^3^ Hematology and Stem Cell Transplant, Ospedali Riuniti Ancona, Ancona, Italy; ^4^ Sorbonne Université, INSERM Centre de Recherche Saint-Antoine, UMRs 938, Service d’hématologie Clinique et de Thérapie Cellulaire, Hôpital Saint Antoine, APHP, Paris, France

**Keywords:** acute myeloid leukemia, allogeneic hematopoietic stem cell transplantation, acute myeloid leukemia relapse, donor lymphocyte infusion (DLI), preemptive therapy

In patients diagnosed with intermediate or high-risk acute myeloid leukemia (AML) allogeneic hematopoietic cell transplantation (allo-HCT) remains in most cases the only curative option. However, despite major improvements in allo-HCT, disease relapse remains the main cause of treatment failure and mortality. AML characteristics (cytogenetic and molecular abnormalities) and disease status, including measurable residual disease level at the time of allo-HCT are well-known risk factors for early AML relapse after transplantation. Nevertheless, it is complex to decipher the respective impact of these parameters and individual prediction of relapse risk remains suboptimal.

In this Research Topic, Zhang et al. develop a nomogram based on these parameters to predict the cumulative incidence of relapse risk after allo-HCT. Such an approach is crucial to identify patients at increased risk of relapse after allo-HCT and may implement some therapeutic strategies to prevent relapse. These include the use of adoptive immunotherapies, i.e. donor lymphocyte infusions (DLI) either alone or in association with chemotherapy or targeted agents. The latter strategies are well summarized in the Review by Ye et al. focusing on the different approaches in the use of DLI (prophylaxis and preemptive but also curative) and their association with other agents such as hypomethylating agents or FLT3-inhibitors. In patients with AML relapse, therapeutic strategy includes the combination of treatment with direct anti-leukemic effect including intensive reinduction therapy, hypomethylating agents and BCL2 inhibitors and immunotherapy with DLI, second allo-HCT but also checkpoint inhibitors. In addition, in patients with particular molecular abnormalities, IDH1/2 inhibitors and FLT3 inhibitors are important options. These different strategies are extensively reviewed in this Research Topic by Webster et al., Abou Dalle et al., and Ciotti et al. Finally, donor choice can also be an important parameter to decrease AML relapse risk after alloHCT. Whether choosing a Haploidentical (Haplo) donor could implement the graft-versus-leukemia effect has not been formally demonstrated yet. However, in this Research Topic Chang et al. comprehensively review the use of Haploidentical donors for AML, allowing the accessibility of allo-HCT to almost all patients. They also report data from recent studies, showing that for some subgroups of AML patients, such as those with measurable residual disease prior to allo-HCT, Haplo-HCT may improve both leukemia-free and overall survival compared to other donor types.

Overall, allo-HCT for AML is a comprehensive platform that must take into account disease characteristics and status prior to transplant to build the most effective strategy to achieve an AML cure starting with the choice of the donor. In the post-transplant setting, close monitoring of measurable residual disease is crucial to driving prophylactic therapeutic strategies and initiating early preemptive treatment to avoid hematological relapse. Finally, even in patients with overt AML relapse, effective therapeutic strategies based on targeted agents and immunotherapy can be offered to the patient to achieve complete remission and AML cure.

## Author Contributions

GB, FS, and FM equally contributed to the Editorial. All authors contributed to the article and approved the submitted version.

## Conflict of Interest

The authors declare that the research was conducted in the absence of any commercial or financial relationships that could be construed as a potential conflict of interest.

## Publisher’s Note

All claims expressed in this article are solely those of the authors and do not necessarily represent those of their affiliated organizations, or those of the publisher, the editors and the reviewers. Any product that may be evaluated in this article, or claim that may be made by its manufacturer, is not guaranteed or endorsed by the publisher.

